# Genomic Insights into Antimicrobial Resistance and Virulence of Monophasic *Salmonella enterica* I 4,[5],12:i:- Isolates from Clinical and Environmental Sources in Jeollanam-do, Korea

**DOI:** 10.3390/microorganisms13122729

**Published:** 2025-11-29

**Authors:** Eunbyeul Go, Bo Ra Kang, Hye Young Na, Hyung Woo Lim, Hye Lin Yang, Mi Young Shin, Yang Joon An, Sook Park, Ki-Bok Yoon

**Affiliations:** Jeollanam-do Institute of Health and Environment, Muan-gun 58568, Jeollanam-do, Republic of Korea; eavygo@korea.kr (E.G.);

**Keywords:** mobile genetic elements (MGEs), antimicrobial resistance genes (ARGs), salmonella pathogenicity islands (SPIs), genotype-phenotype concordance, molecular epidemiology

## Abstract

This study investigated the molecular epidemiology, virulence, antimicrobial resistance, and mobile genetic elements (MGEs) of *Salmonella enterica* serovar I 4,[5],12:i:- isolates collected in Jeollanam-do, South Korea, between 2021 and 2023. A total of 135 isolates were tested for antimicrobial susceptibility and 14 virulence-associated genes were screened by PCR. Pulsed-field gel electrophoresis (PFGE) assessed clonal relatedness, and whole-genome sequencing (WGS) enabled multilocus sequence typing (MLST), core genome MLST (cgMLST), SNP phylogeny, resistance gene detection, and MGE analysis. Nine virulence profiles (VP1–VP9) were identified. VP1 (74.1%) was strongly associated with multidrug resistance (MDR), while VP2 (14.8%), which carried plasmid-encoded *spv* genes, remained largely susceptible. Overall, 83.7% of isolates were resistant to at least one antimicrobial, and 65.2% were MDR, with ampicillin and tetracycline consistently forming the backbone of MDR phenotypes. PFGE revealed high genetic diversity, with 72 pulsotypes, yet certain clones (e.g., SMOX01.006, SMOX01.012) were widely distributed and corresponded to VP2 isolates. WGS confirmed two dominant sequence types, ST34 (*n* = 24) and ST19 (*n* = 20), with SNP phylogeny showing VP1 isolates mainly clustered with ST34 and VP2 with ST19. Genotype–phenotype concordance showed strong agreement for most antimicrobials, except cefoxitin, ciprofloxacin, amikacin, and trimethoprim/sulfamethoxazole. MGE analysis revealed that *tet(B)* was consistently associated with ISVsa5, while ISEc59 was linked to multiple resistance genes, though only *aac(3)-IV* was phenotypically expressed. These findings demonstrate that MDR and virulence gene composition were closely associated with clonal clustering and that MGEs may contribute to resistance gene expression. This study provides a basis for understanding the dissemination of resistant and virulent *Salmonella* in the region and underscores the need for continuous genomic surveillance.

## 1. Introduction

Non-typhoidal *Salmonella* is a leading cause of foodborne illnesses worldwide and a zoonotic pathogen with a broad host range, including humans, swine, cattle, and poultry [1,2,3]. Recent global estimates indicate that approximately 150 million human infections and 60,000 deaths are attributable to *Salmonella* each year [4]. Numerous studies have investigated the epidemiological study, genomic features, virulence mechanisms, host-pathogen interactions and antimicrobial resistance patterns of various *Salmonella* serotypes [5,6,7,8,9]. Among the diverse serotypes, *Salmonella enterica* serovars Typhimurium and Enteritidis are recognized for their low host specificity and high pathogenic potential, enabling them to spread rapidly across diverse hosts and environments [10]. Given their widespread distribution, understanding the virulence mechanisms of these serovars is essential. The pathogenicity of *Salmonella* is primarily mediated by *Salmonella* pathogenicity islands (SPIs) and plasmid-encoded virulence genes, both of which play essential roles in bacterial invasion, intracellular survival, and systemic dissemination. Notably, SPIs often harbor genes associated not only with virulence but also with antimicrobial resistance, indicating a functional association between pathogenicity and resistance mechanisms [11,12].

Recently, particular attention has been given to *Salmonella enterica* serovar I 4,[5],12:i:-, a monophasic variant of *S*. Typhimurium, which has emerged as a major public health concern due to its association with widespread outbreaks and increasing antimicrobial resistance [13,14,15]. This variant is characterized by the loss of the second-phase flagellin gene (*fljB*), resulting in a monophasic phenotype that may facilitate immune evasion and persistence within hosts [16]. In Korea, *S*. I 4,[5],12:i:- has been reported as the third most prevalent serovar, frequently isolated from human patients, animals, food and environmental sources such as wastewater. Although several studies have addressed the epidemiology of *S*. I 4,[5],12:i:- globally, molecular epidemiological investigations focusing on isolates from Korea, particularly utilizing high-resolution genomic analyses, remain limited. Therefore, in this study, we aimed to analyze the molecular epidemiological characteristics of *S*. I 4,[5],12:i:- isolates collected from various sources in Jeollanam-do, Korea. This study was conducted to elucidate how *Salmonella* isolates collected in Jeollanam-do are grouped into specific clonal lineages, to assess their genetic relatedness, and to characterize their antimicrobial susceptibility profiles through molecular epidemiological analysis.

## 2. Materials and Methods

### 2.1. Bacterial Isolates

**Figure 1:**
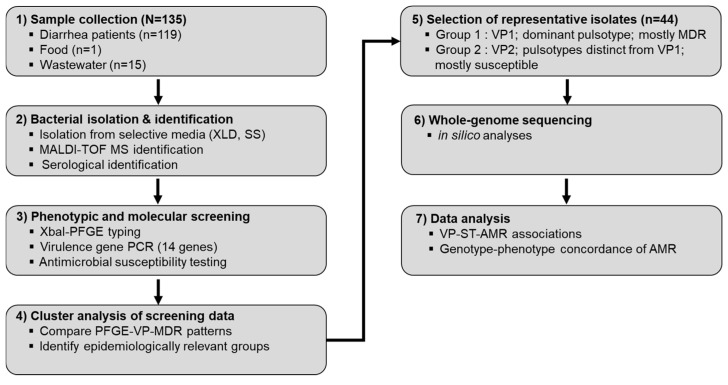
Schematic overview of the workflow used in this study.

A total of 135 *S*. I 4,[5],12:i:- strains were analyzed in this study. These strains were previously isolated and preserved from various sources in Jeollanam-do, South Korea. The isolates originated from samples collected between 2021 and 2023, including acute diarrhea patients (*n* = 119), swine (*n* = 1), and wastewater (*n* = 15). For primary isolation, samples were streaked onto selective agar plates (XLD and SS agar), and colonies exhibiting black pigmentation were sub-cultured on Tryptic soy agar (TSA). Bacterial identification was performed using the MALDI-TOF Biotyper (Bruker Daltonics GmbH, Bremen, Germany) and serological testing. Serovars were identified according to the Kauffmann-White classification scheme. Ethical approval was not required as all samples were anonymized and not personally identifiable. The subsequent experimental procedures followed the study workflow illustrated in Figure 1.

### 2.2. Detection of Virulence Genes

Virulence genes were detected by PCR using DNA templates prepared by boiling method. Fourteen virulence-associated genes were screened, including *Salmonella* pathogenicity island genes (*invA*, *sseL*, *mgtC*, *siiE*, *sopB*), plasmid-encoded virulence genes (*spvB*, *spvC*, *spvR*, *pefA*), and prophage-associated virulence genes (*gipA*, *gtgB*, *sopE*, *sspH1*, *sspH2*), using primers and PCR conditions described in previous study [17]. Each 20 µL mixture consisted of 10 µL of Quick Taq^®^ HS DyeMix (Toyobo, Osaka, Japan), 10 pmol of each primer, and 5 µL of template. PCR conditions were 94 °C for 10 min, 35 cycles of 94 °C for 30 s, 58 °C for 30 s, and 72 °C for 1 min, followed by 72 °C for 7 min.

### 2.3. Antimicrobial Susceptibility Testing

Antimicrobial susceptibility testing was performed using the broth microdilution method with the KRCDC2F plate (Thermo Fisher Scientific, Waltham, MA, USA), which includes 15 antimicrobial agents: ampicillin, imipenem, cefotaxime, ceftriaxone, ceftazidime, cefoxitin, tetracycline, chloramphenicol, trimethoprim/sulfamethoxazole, nalidixic acid, ciprofloxacin, gentamicin, amikacin, azithromycin, and colistin. Fresh colonies were suspended in 0.85% NaCl to a turbidity equivalent to a 0.5 McFarland standard. A 10 µL aliquot was inoculated into 11 mL of cation-adjusted Mueller-Hinton broth. Then, 50 µL of the inoculum was dispensed into each well. Plates were incubated at 37 °C for 18 h. Minimum inhibitory concentrations (MICs) were determined and interpreted according to the Clinical and Laboratory Standards Institute (CLSI) guidelines (M100-Ed34, 2024) [18]. Multidrug resistance (MDR) was defined as acquired resistance to at least one agent in three or more antimicrobial categories [19].

### 2.4. Pulsed-Field Gel Electrophoresis (PFGE)

PFGE was conducted according to the standardized PulseNet International protocol (https://www.pulsenetinternational.org (accessed on 10 August 2024)) using the CHEF-Mapper XA system (Bio-Rad, Hercules, CA, USA). Bacterial cultures grown on tryptic soy agar were suspended in cell suspension buffer (100 mM Tris, 100 mM EDTA, pH 8.0) and embedded in 1.2% agarose plugs. The plugs were digested with XbaI for 5 h at 37 °C. Electrophoresis conditions included an initial switch time of 2.16 s, a final switch time of 63.8 s, and a total run time of 18 h. *S.* Braenderup BAA-664 was used as a size marker. PFGE patterns were analyzed using BioNumerics v4.61 (Applied Maths, Sint-Martens-Latem, Belgium). Similarity was calculated using the Dice coefficient, and cluster analysis was performed using the unweighted pair group method with arithmetic mean (UPGMA).

### 2.5. Whole-Genome Sequencing and In Silico Analysis

To ensure that the genomic analysis reflected the major epidemiological features, 44 representative isolates were selected for WGS. These included all VP2 isolates, which clustered independently from VP1, and VP1 isolates from the largest pulsotype group, representing the predominant clonal population in PFGE. Genomic DNA was extracted using the QIAamp DNeasy PowerSoil Kit (Qiagen, Hilden, Germany). DNA purity and concentration were assessed using both a NanoDrop spectrophotometer (Thermo Fisher Scientific, Waltham, MA, USA) and the Qubit™ 4 Fluorometer (Thermo Fisher Scientific, Waltham, MA, USA). Sequencing libraries were prepared using the Illumina DNA Prep Tagmentation kit (Illumina, San Diego, CA, USA) according to the manufacturer’s instructions. Whole-genome sequencing was performed on an Illumina MiSeq platform (Illumina, San Diego, CA, USA) to generate 150-bp paired-end reads. On average, 1.3 million reads were produced per sample and the quality of raw reads was evaluated using FastQC (v0.12.1) [20]. Raw sequences were trimmed and de novo assembled using CLC Genomics Workbench (Qiagen, Hilden, Germany), resulting in an average sequencing coverage depth of approximately 38×. Genome completeness was assessed using BUSCO (v6.0.0), and all assemblies exhibited ≥97.6% completeness [21]. All assemblies used in this study satisfied quality criteria, including assembly size of 4.89–5.27 Mbp, N50 > 140 kb, and ≤110 contigs.

After quality filtering and assembly validation, the assemblies were subsequently used for core genome multilocus sequence typing (cgMLST), single-nucleotide polymorphism (SNP)-based subtyping, antimicrobial resistance gene (ARG) and mobile genetic elements (MGEs) detection. These genomic analyses were performed using cgMLSTFinder v1.0.1, CSIPhylogeny 1.4, ResFinder v4.7.2, MobileElementFinder v1.0.3 [22] provided by the Center for Genomic Epidemiology (https://cge.food.dtu.dk/ (accessed on 1 May 2025)). The genome of *S*. *enterica* serovar Typhimurium strain LT2 (NCBI RefSeq: NC_003197.2) was used as a reference for SNP calling. A SNP-based phylogenetic tree was generated in Newick format and visualized along with associated datasets using the Interactive Tree of Life (iTOL) v7.

### 2.6. Data Analysis

Associations between virulence profiles and antimicrobial resistance profiles were evaluated using Fisher’s exact test. A significance level of *p* < 0.05 was considered statistically significant. Concordance between phenotypic and genotypic antimicrobial resistance was assessed by Cohen’s kappa (ĸ), sensitivity, specificity, positive predictive value (PPV), and negative predictive value (NPV). All statistical analyses were performed using R software (v4.5.1).

## 3. Results

### 3.1. Virulence Gene Distribution

Nine distinct virulence gene profiles (VP1-VP9) were identified among the *S*. I 4,[5],12:i:- isolates based on the presence or absence of 14 virulence-associated genes. All isolates harbored *Salmonella* pathogenicity island (SPI) genes (*invA*, *sseL*, *mgtC*, *siiE*, and *sopB*), which are essential for host cell invasion and intracellular survival. In addition, most isolates carried *gipA*, *gtgB*, and *sspH2*, indicating the presence of prophage-derived virulence factors involved in intestinal colonization and immune evasion. This suggests that these isolates possess not only SPI-associated virulence attributes but also prophage-mediated pathogenicity. The most prevalent profile, VP1 (*invA-sseL-mgtC-siiE-sopB-gipA-gtgB-sspH2*), was observed in 100 isolates (74.1%). VP2 (14.8%) was distinguished by the presence of plasmid-associated virulence genes (*spvB*, *spvC*, *spvR*, and *pefA*), which are typically linked to systemic dissemination and increased pathogenic potential. The remaining profiles (VP3-VP9) were identified in only 1 to 4 isolates each (0.7–3.0%), and their virulence gene profiles were classified based on the distribution of prophage-encoded virulence genes. Only VP6 harbored *sspH1* gene, and two isolates (VP7–VP8) carried *sopE* gene, as shown in Table 1. For the 44 isolates subjected to WGS, all virulence genes detected by PCR were also confirmed by WGS, indicating complete concordance between both methods.

### 3.2. Antimicrobial Resistance Phenotype of S. I 4,[5],12:i:- Isolates

A total of 135 *S*. I 4,[5],12:i:- isolates were tested for antimicrobial susceptibility. Among them, 22 isolates (16.3%) were susceptible to all tested antibiotics. The remaining 113 isolates (83.7%) showed resistance to at least one agent. The most common single-drug resistances were to ampicillin (7.4%), tetracycline (3.7%), and nalidixic acid (2.2%). Multidrug resistance (MDR), defined as resistance to ≥3 antimicrobial classes, was observed in 85 isolates (63.0%). The most prevalent MDR profile, comprising resistance to cefotaxime, ampicillin, ceftriaxone, tetracycline, chloramphenicol, and ceftazidime was detected in 47 isolates (34.8%). Additional MDR patterns included combinations involving quinolones, aminoglycosides, sulfonamides, and macrolides, contributing to a diverse range of resistance profiles among the isolates (Table 2).

**Figure 2:**
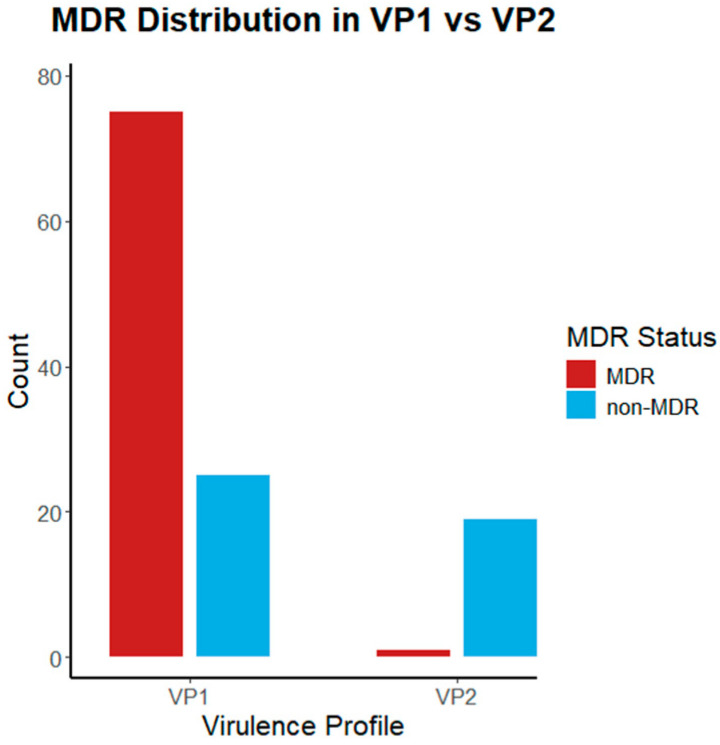
Distribution of MDR (red) and non-MDR (blue) isolates between the two predominant virulence profiles (VP1 and VP2). Notably, VP2 contained only one MDR, with all remaining isolates being susceptible. The bar plot was generated using R software.

A significant association was observed between two major virulence types (VP1 and VP2) and antimicrobial resistance phenotypes (MDR and non-MDR). According to Fisher’s exact test (*p* < 0.001), the distribution of VP1 and VP2 differed markedly across resistance categories. VP1 was primarily associated with MDR isolates, whereas VP2 appeared more frequently among non-MDR isolates. These differences in proportional distribution indicate that virulence profile composition varies according to resistance status (Figure 2).

### 3.3. PFGE Analysis and Representative Isolate Selection

**Figure 3:**
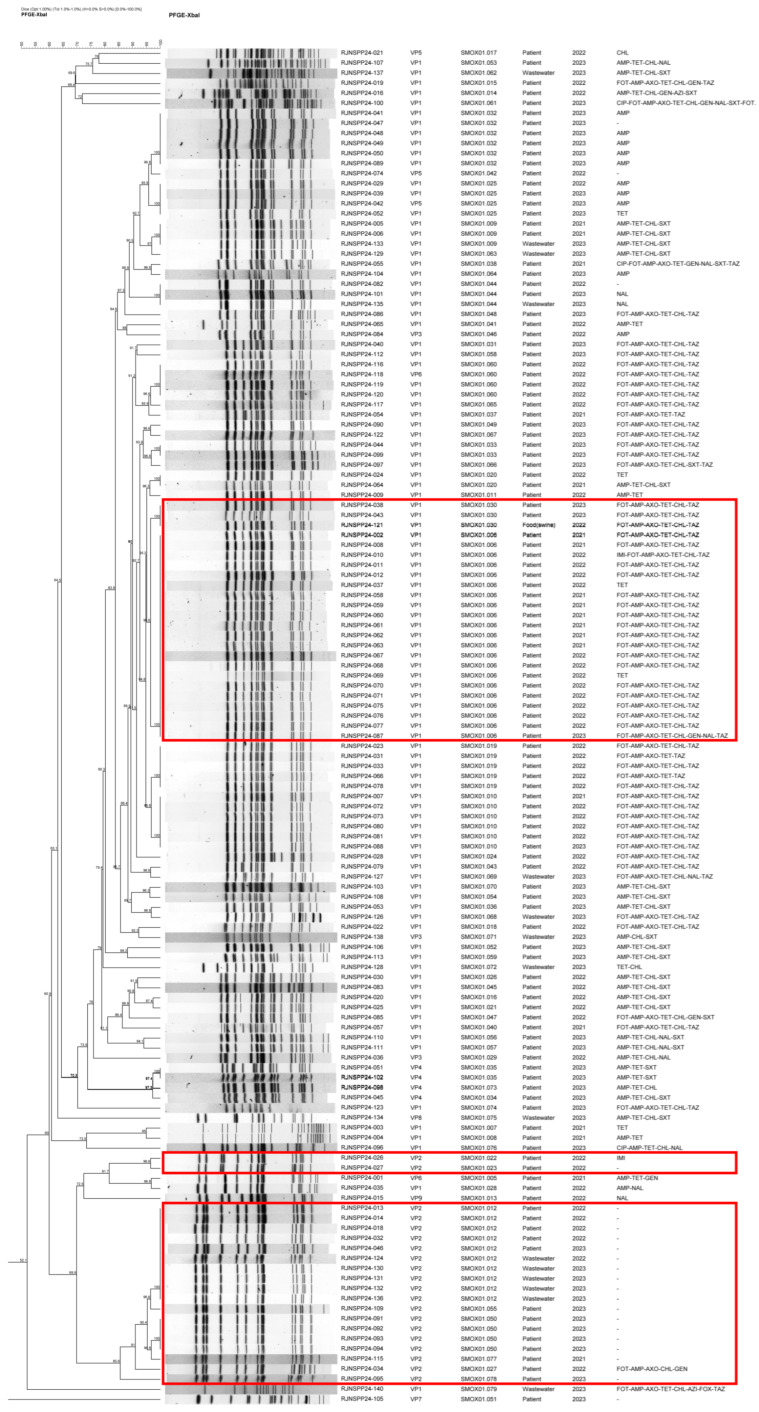
PFGE dendrogram of *S.* I,4,[5],12:i:- isolates with associated virulence profiles and resistant patterns. The dendrogram was generated from XbaI-digested patterns analyzed using the Dice coefficient and clustered by UPGMA. Virulence profiles (VP1–VP9) and resistant patterns are displayed alongside each isolate. Forty-four isolates subjected to whole-genome sequencing are indicated with red boxes.

PFGE analysis revealed that the 135 isolates were classified into 74 distinct pulsotypes, demonstrating substantial genetic diversity among the isolates. (Figure 3). The most prevalent pulsotype was SMOX01.006 (15.6%), which belonged to VP1, followed by SMOX01.012 (7.4%), which belonged to VP2. VP1 isolates showed a wider pulsotype distribution, which reflected their higher representation in the collection, whereas VP2 isolates, represented in smaller numbers, were concentrated within fewer pulsotypes.

Notably, more than half of the VP2 isolates clustered within the SMOX01.012 pulsotype or closely related pulsotypes, forming a discrete PFGE grouping. In contrast, VP1 isolates were dispersed across multiple clusters with greater pulsotype heterogeneity. PFGE clustering also showed that VP2 isolates harboring plasmid-associated virulence genes formed independent molecular clusters that corresponded with their virulence gene profiles. Based on the PFGE clustering patterns, representative isolates were selected by integrating information on isolation source, antimicrobial susceptibility profiles, and VP types to identify strains exhibiting characteristic features within major pulsotype groups.

### 3.4. Genomic Typing and Clonal Relationships

**Figure 4:**
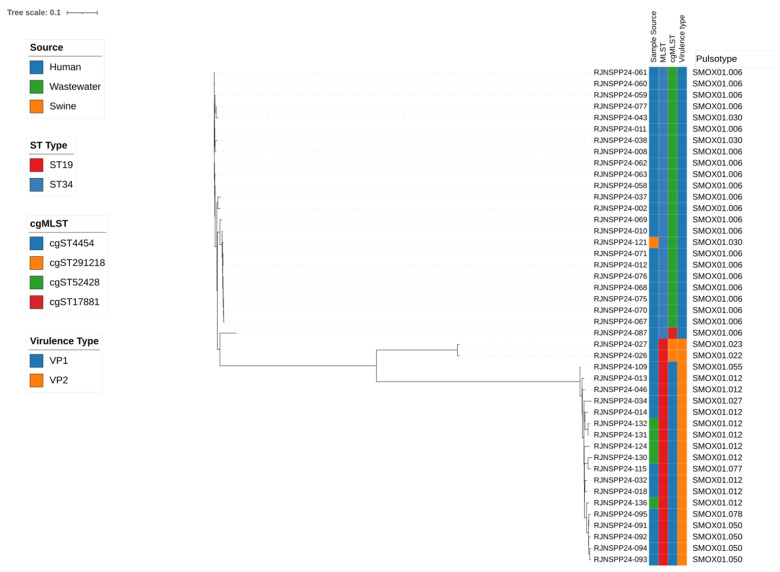
Core genome SNP-based phylogenetic tree of *S.* I 4,[5],12:i:- isolates annotated with source, sequence type (ST), virulence profile, and pulsotype. ST34 and ST19 isolates formed distinct phylogenetic clusters, with minimal SNP differences observed within each cluster, forming clearly separated clonal groups.

To further resolve the molecular differences among the selected isolate groups, whole-genome sequence-based typing was performed. MLST analysis identified two predominant sequence types: ST34 (*n* = 24) and ST19 (*n* = 20). According to cgMLST analysis, the isolates were further classified into four distinct profiles: cgST52428 (*n* = 23), cgST4454 (*n* = 18), cgST291218 (*n* = 2), and cgST17881 (*n* = 1). Overall, isolates that were closely related according to MLST were also grouped similarly by cgMLST, indicating that genetically similar strains were consistently identified across both typing approaches (Supplementary Table S1). The majority of ST34 isolates belonged to cgST52428, while most ST19 isolates were associated with cgST4454. Core genome SNP analysis revealed that isolates sharing the same cgMLST type clustered closely in the phylogenetic tree. Isolates within each cgST exhibited minimal SNP differences (typically ≤ 20 SNPs), suggesting that they were distributed within genetically similar clonal lineages. In contrast, isolates belonging to different cgMLST types were separated by over 100 SNPs, indicating more distant genetic relationships. Although the number of isolates from each source was insufficient for meaningful comparative analysis, the overall distribution patterns were mainly differentiated by VP and ST type rather than by source (Figure 4).

### 3.5. Concordance Between Antimicrobial Resistance Phenotype and Genotype

Genotype-phenotype concordance was assessed based on resistance determinants identified by ResFinder and AST results (Table 3). Overall, agreement levels varied substantially across antimicrobial classes. β-lactams, including cefotaxime, ampicillin, ceftriaxone, and ceftazidime, showed almost perfect agreement (ĸ = 0.95–1.00), with complete concordance between genes and phenotypic resistance. Tetracycline and chloramphenicol likewise demonstrated almost perfect concordance (ĸ = 0.95–1.00), supported by the consistent detection of corresponding genes in resistant isolates.

In contrast, quinolones, aminoglycosides, and trimethoprim-sulfamethoxazole exhibited poor agreement (ĸ = 0.00–0.05). For these agents, phenotypic resistance frequently occurred in the absence of the corresponding resistance genes, indicating substantial genotype-phenotype discrepancies. Gentamicin showed particularly low concordance, although multiple resistance genes were detected. These findings suggest that the low concordance observed in these antimicrobial classes may reflect resistance mechanisms mediated by multiple pathways rather than genetic factors.

**Figure 5:**
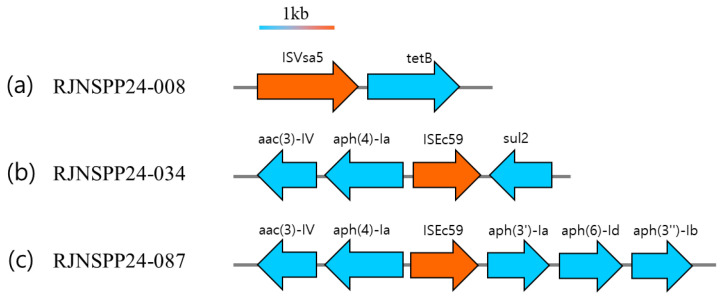
Arrangement of ARGs and IS elements on representative contigs. Schematic maps illustrate the genetic context and organization of antimicrobial resistance genes (ARGs) and insertion sequences (ISs). Arrows represent genes or mobile elements; ARGs are shown in blue and IS elements in orange. The scale bar indicates the approximate base pairs of genes and mobile elements. (**a**) Genetic map of a representative isolate carrying ISVsa5 and the *tet(B)* gene. (**b**,**c**) Genetic maps of isolates harboring ISEc59 and the surrounding antimicrobial resistance genes.

Sequence analysis of mobile elements and ARGs revealed consistent genomic arrangements among tetracycline-resistant isolates. Nearly all tetracycline-resistant isolates carried the *tet(B)* gene and the ISVsa5 insertion sequence on the same contig, whereas isolates lacking *tet(B)* contained only ISVsa5 without a corresponding resistance gene (Figure 5a). In addition, two gentamicin-resistant isolates carried the *aac(3)-IV* gene, responsible for resistance to gentamicin and tobramycin, together with the ISEc59 mobile element on the same contig (Figure 5b,c). Although multiple ARGs were present within the contig regions containing ISEc59, only *aac(3)-IV* corresponded to the observed phenotypic resistance. These findings show that specific IS elements were detected on the same contigs as their corresponding resistance genes in the examined isolates.

## 4. Discussion

Our study provides insights into virulence and resistance characteristics of *S. enterica* serovar I 4,[5],12:i:- isolates collected in Jeollanam-do, Korea, between 2021 and 2023. Virulence data showed that all isolates carried SPIs, which are essential not only for establishing pathogenicity but also for enabling *Salmonella* to survive within the host. Previous studies have demonstrated that the lack of specific SPI genes exhibits impaired intracellular survival and fails to cause disease, underscoring the indispensable role of SPIs in *Salmonella* pathogenesis [23]. In contrast, plasmid-mediated virulence genes, particularly *spv* genes, provided discriminatory power for differentiating the isolates in our study, representing a key feature that distinguished the strains under investigation.

Antimicrobial susceptibility patterns further reflected this distinction. With the exception of two isolates, VP2 strains carrying plasmid-borne *spv* genes remained largely susceptible to the tested antibiotics, whereas isolates belonging to other virulence profiles exhibited diverse resistance patterns. In our study, a total of 85 isolates (63.0%) were classified as MDR, with the predominant resistance pattern being FOT-AMP-AXO-TET-CHL-TAZ (34.8%). Although a subset of isolates exhibited resistance only to ampicillin and tetracycline, these two antimicrobials were consistently observed across nearly all MDR patterns. This finding suggests that ampicillin and tetracycline resistance constitute fundamental traits underlying the MDR phenotypes in the population. Consistent with our results, several recent studies have also reported high rates of ampicillin and tetracycline resistance in *S.* I 4,[5],12:i:- [24,25,26]. In particular, some studies noted that tetracycline resistance was frequently observed together with resistance to other antimicrobials, indicating its common presence within broader MDR patterns [27,28].

PFGE analysis classified the isolates 72 pulsotypes, indicating substantial genetic diversity. Nevertheless, the predominance of specific clones, such as SMOX01.006 and SMOX01.012, suggests that certain lineages were widely distributed in the population. These pulsotypes corresponded to VP2 isolates carrying plasmid-mediated virulence genes, which formed an independent cluster. This finding implies that virulence gene composition may be associated with molecular clustering among the isolates. Interestingly, isolates representing VP1 and VP2 pulsotypes that were subjected to WGS clustered consistently with sequence type classification and SNP-based phylogeny. For example, VP1 isolates were predominantly associated with ST34, whereas VP2 isolates corresponded to ST19. This concordance demonstrates that PFGE-based clustering and WGS analyses both reflected the underlying genomic population structure. This indicates that virulence gene composition, particularly the presence of plasmid-mediated *spv* genes in VP2 isolates, is associated with the formation of distinct molecular clusters. Similar findings have been reported in previous studies, in which *Salmonella* isolates of the same serovar clustered into genetically distinct clades according to their virulence factor [29].

The low concordance observed for cefoxitin, quinolones, aminoglycosides, and sulfonamides is unlikely to be explained simply by the absence of known resistance genes. Instead, it may reflect the involvement of multiple resistance mechanisms, including increased efflux pump activity, chromosomal mutations, or regulatory changes that influence antimicrobial susceptibility [30]. In particular, fluoroquinolone resistance has been shown to arise from stepwise mutations in chromosomal genes such as *gyrA* and *parC*, often together with *qnr* determinants, which can collectively contribute to high-level resistance [31]. Therefore, phenotypic resistance to these antimicrobial classes cannot be fully inferred from gene presence alone, underscoring the need for additional molecular investigations, such as expression or mutation analyses, to better characterize the underlying resistance mechanisms.

All isolates carrying the *tet(B)* gene exhibited resistance to tetracycline, and this gene was consistently found in association with the ISVsa5 element. In contrast, isolates harboring ISVsa5 alone remained susceptible to tetracycline. Moreover, strains carrying the ISEc59 element contained multiple resistance genes, but only *aac(3)-IV* was phenotypically expressed. These findings suggest that the association with insertion sequences may contribute to the expression of *tet(B)* and other antimicrobial resistance genes.

This study demonstrated that monophasic *Salmonella* isolates from Jeollanam-do could be divided into two major clonal lineages. One lineage was characterized by multidrug resistance and the presence of diverse resistance genes but lacked *spv* genes, the plasmid-mediated virulence genes, whereas the other lineage carried *spv* genes but remained largely susceptible to antimicrobials. This mutually exclusive pattern between antimicrobial resistance and *spv* carriage was consistent with sequence type and SNP-based clustering, suggesting a trade-off in survival strategies. Similar findings have been reported in recent studies, supporting the relevance of our results [32].

Most importantly, our results showed that the plasmid-mediated quinolone resistance gene *qnrS1* was detected, while no chromosomal mutations in *gyrA* or *parC* were identified based on ResFinder analysis. Notably, 95% of *qnrS1*-positive isolates exhibited reduced susceptibility to ciprofloxacin (Supplementary Table S2), indicating that low-level resistance conferred by *qnrS1* alone may contribute to phenotypic resistance even in the absence of QRDR mutations. These findings are consistent with emerging global trends, as highlighted in the 2024 WHO Bacterial Priority Pathogens List (BPPL), which classifies fluoroquinolone-resistant *Salmonella* and *Shigella* among high-priority pathogens due to increasing resistance rates, limited treatment options, and significant public health impact. The observation of reduced susceptibility associated with *qnrS1* in our isolates underscores the clinical and epidemiological relevance of plasmid-mediated fluoroquinolone resistance and aligns with WHO’s concerns regarding the continued spread of fluoroquinolone resistance determinants [33].

Unlike prior studies that only described general associations between virulence genes and antimicrobial resistance, our results show that isolates carrying SPIs and plasmid-mediated virulence genes form a susceptible group, whereas other virulence profiles are linked to diverse MDR patterns. This indicates a distinct insight previously not reported.

This study has several limitations. First, most isolates were derived from clinical cases, which may not fully represent *Salmonella* circulating in non-human reservoirs. Second, resistance gene analysis was restricted to determinants corresponding to the antimicrobial classes evaluated phenotypically. Third, although virulence gene profiles were well characterized, corresponding virulence phenotypes were not experimentally validated. Future studies should expand WGS-based surveillance to diverse sources to clarify transmission dynamics and cross-reservoir dissemination. Additional molecular investigations, such as gene expression and mutation analyses, will be essential to elucidate genotype-phenotype discrepancies, particularly for antimicrobial classes driven by multiple pathways. Experimental evaluation of virulence phenotypes will further strengthen the epidemiology of monophasic *Salmonella* Typhimurium.

In conclusion, this study characterized the antimicrobial resistance and virulence traits of *S.* I 4,[5],12:i:- isolates circulating in Jeollanam-do, a major pathogen responsible for persistent foodborne infections. This study provides a basis for future studies on the sources and transmission of *Salmonella* and serves as a foundation for strengthening public health surveillance.

## Figures and Tables

**Table 1:** Virulence gene profiles (VPs) of the *S.* I 4,[5],12:i:- isolates. Virulence gene profiles were categorized based on the presence of *Salmonella* pathogenicity island genes, plasmid-encoded virulence genes, and prophage-associated virulence genes. Each VP represents a distinct combination of these genes in individual isolates, allowing comparison of major virulence gene patterns across the population. “+”, gene presence; “-”, gene absence.

VP	*Salmonella* Pathogenicity Island Genes					Plasmid Virulence Genes				Prophage Virulence Genes					No. (%)
	*invA*	*sseL*	*mgtC*	*siiE*	*sopB*	*spvB*	*spvC*	*spvR*	*pefA*	*gipA*	*gtgB*	*sopE*	*sspH1*	*sspH2*	
VP1	+	+	+	+	+	-	-	-	-	+	+	-	-	+	100 (74.1)
VP2	+	+	+	+	+	+	+	+	+	+	+	-	-	+	20 (14.8)
VP3	+	+	+	+	+	-	-	-	-	-	+	-	-	+	3 (2.2)
VP4	+	+	+	+	+	-	-	-	-	+	-	-	-	+	4 (3.0)
VP5	+	+	+	+	+	-	-	-	-	+	+	-	-	-	3 (2.2)
VP6	+	+	+	+	+	-	-	-	-	+	+	-	+	+	2 (1.5)
VP7	+	+	+	+	+	-	-	-	-	+	-	+	-	-	1 (0.7)
VP8	+	+	+	+	+	-	-	-	-	-	+	+	-	+	1 (0.7)
VP9	+	+	+	+	+	+	+	+	+	-	+	-	-	+	1 (0.7)

**Table 2:** Antimicrobial resistance profiles among *S.* I 4,[5],12:i:- isolates. Resistance profiles were categorized based on the number of antimicrobial classes to which each isolate exhibited non-susceptibility. “Susceptible” indicates isolates showing no phenotypic resistance to the tested agents. “Resistance” represents isolates resistant to one or two antimicrobial agents belonging to a single or two classes. “MDR” indicates resistance to three or more antimicrobial classes.

Type of Resistance	Antimicrobial Resistance Profile	No. of Isolates	% of Isolates	No. of AMR Classes
Susceptible	None	22	16.3	0
Resistance	AMP	10	7.4	1
	CHL	1	0.7	1
	IMI	1	0.7	1
	NAL	3	2.2	1
	TET	5	3.7	1
	AMP-NAL	1	0.7	2
	AMP-TET	3	2.2	2
	TET-CHL	1	0.7	2
	FOT-AMP-AXO-TET-TAZ	3	2.2	2
MDR	AMP-CHL-SXT	1	0.7	3
	AMP-TET-CHL	1	0.7	3
	AMP-TET-GEN	1	0.7	3
	AMP-TET-SXT	2	1.5	3
	AMP-TET-CHL-NAL	2	1.5	4
	AMP-TET-CHL-SXT	17	12.6	4
	AMP-TET-CHL-NAL-SXT	2	1.5	5
	CIP-AMP-TET-CHL-NAL	1	0.7	4
	FOT-AMP-AXO-CHL-GEN	1	0.7	3
	AMP-TET-CHL-GEN-AZI-SXT	1	0.7	6
	FOT-AMP-AXO-TET-CHL-TAZ	47	34.8	3
	FOT-AMP-AXO-TET-CHL-GEN-SXT	1	0.7	6
	FOT-AMP-AXO-TET-CHL-GEN-TAZ	1	0.7	4
	FOT-AMP-AXO-TET-CHL-NAL-TAZ	1	0.7	4
	FOT-AMP-AXO-TET-CHL-SXT-TAZ	1	0.7	5
	IMI-FOT-AMP-AXO-TET-CHL-TAZ	1	0.7	3
	FOT-AMP-AXO-TET-CHL-AZI-FOX-TAZ	1	0.7	4
	FOT-AMP-AXO-TET-CHL-GEN-NAL-TAZ	1	0.7	5
	CIP-FOT-AMP-AXO-TET-GEN-NAL-SXT-TAZ	1	0.7	5
	CIP-FOT-AMP-AXO-TET-CHL-GEN-NAL-SXT-FOT-TAZ	1	0.7	6

AMP: ampicillin; CHL: chloramphenicol; IMI: imipenem; NAL: nalidixic acid; TET: tetracycline; SXT: trimethoprim/sulfamethoxazole; GEN: gentamicin, CIP: ciprofloxacin; FOT: cefotaxime; AXO: ceftriaxone; AZI: azithromycin; FOX: cefoxitin; TAZ: ceftazidime.

**Table 3:** Concordance between antimicrobial resistance genes and phenotypic susceptibility results across tested antibiotic agents. The concordance between detected resistance genes (genotype) and phenotypic antimicrobial susceptibility results for each antibiotic. ResFinder-derived resistance genes corresponding to the antimicrobial classes tested in the antimicrobial susceptibility testing were selected and schematized to facilitate genotype-phenotype comparison across antibiotics.

Antibiotics		Resistance Gene (ResFinder)	*n*	TP	TN	FP	FN	ĸ	Sensitivity (%)	Specificity (%)	PPV (%)	NPV (%)	Accuracy (%)	Agreement Level
β-lactams	FOT	*bla*_CTX-M_, *bla*_OXA_	44	23	21	0	0	1.00	100.0	100.0	100.0	100.0	100.0	Almost Perfect
	AMP	*bla*_CTX-M_, *bla*_OXA_	44	23	21	0	0	1.00	100.0	100.0	100.0	100.0	100.0	Almost Perfect
	AXO	*bla*_CTX-M_, *bla*_OXA_	44	23	21	0	0	1.00	100.0	100.0	100.0	100.0	100.0	Almost Perfect
	FOX	*bla*_CTX-M_, *bla*_OXA_	44	0	21	23	0	0.00	NA	47.7	0.0	100.0	47.7	Poor
	TAZ	*bla*_CTX-M_, *bla*_OXA_	44	22	21	1	0	0.95	100.0	95.5	95.7	100.0	97.7	Almost Perfect
Quinolones	CIP	*qnrS1*	44	0	22	22	0	0.00	NA	50.0	0.0	100.0	50.0	Poor
	NAL	*qnrS1*	44	1	22	21	0	0.05	100.0	51.2	4.5	100.0	52.3	Poor
Tetracycline	TET	*tet(A)*, *tet(B)*	44	22	21	1	0	0.95	100.0	95.5	95.7	100.0	97.7	Almost Perfect
Phenicols	CHL	*floR*, *catA2*, *cmlA1*	44	23	21	0	0	1.00	100.0	100.0	100.0	100.0	100.0	Almost Perfect
Aminoglycosides	GEN	*aac(6′)-Iaa*, *aac(3)-IV*, *aph*	44	2	0	42	0	0.00	100.0	0.0	4.5	0.0	4.5	Poor
	AMI	*aac(3)-IV*, *aph*	44	0	42	2	0	0.00	NA	95.5	0.0	100.0	95.5	Poor
Sulfonamides	SXT	*sul2*, *dfrA14*	44	0	41	3	0	0.00	NA	93.2	0.0	100.0	93.2	Poor

Statistical measurement: TP, true positive; TN, true negative; FP, false positive; FN, false negative; PPV, Positive predictive value; NPV, Negative predictive value; NA, Not applicable. Antimicrobial agents: FOT, Cefotaxime; AMP, Ampicillin; AXO, Ceftriaxone; FOX, Cefoxitin; TAZ, Ceftazidime; CIP, Ciprofloxacin; NAL, Nalidixic acid; TET, Tetracycline; CHL, Chloramphenicol; GEN: Gentamicin; AMI: Amikacin; SXT: Trimethoprim/Sulfamethoxazole. Agreement interpretation: ĸ < 0.00 Poor; 0.81 ≤ ĸ ≤ 1.00 Almost Perfect.

## Data Availability

The data that support the findings will be available in NCBI GenBank at https://www.ncbi.nlm.nih.gov/bioproject/PRJNA1272471 (accessed on 26 November 2025) following an embargo from the date of publication to allow for commercialization of research findings.

## Supplementary Materials

The following supporting information can be downloaded at: https://www.mdpi.com/article/10.3390/microorganisms13122729/s1, Table S1: MLST and cgMLST types and NCBI BioSample accession numbers of isolates. Table S2. Antimicrobial susceptibility profiles of qnrS1-positive isolates. The table presents MIC values and categorical interpretations for each antimicrobial class.
