# Supplementary material for: Genomic Insights into Antimicrobial Resistance and Virulence of Monophasic Salmonella enterica I 4,[5],12:i:- Isolates from Clinical and Environmental Sources in Jeollanam-do, Korea
Source: Microorganisms. 2025 Nov 29;13(12):2729. doi: 10.3390/microorganisms13122729 (PMC12735187; doi:10.3390/microorganisms13122729)
Supplement: Supplementary file 1 [file microorganisms-13-02729-s001.zip › microorganisms-3934351-supplementary.pdf]

Table S1. Summary of the source, collection year, MLST, and cgMLST profiles, and the associated NCBI BioSample and SRA accession numbers for each isolates

Strain ID	Source	Collection Year	MLST	cgMLST	BioSample accession	SRA accession number
RJNSPP24-002	Human	2021	ST34	cgST52428	SAMN48909850	SRR36174788
RJNSPP24-008	Human	2021	ST34	cgST52428	SAMN48909851	SRR36174787
RJNSPP24-010	Human	2022	ST34	cgST52428	SAMN48909852	SRR36174776
RJNSPP24-011	Human	2022	ST34	cgST52428	SAMN48909853	SRR36174765
RJNSPP24-012	Human	2022	ST34	cgST52428	SAMN48909854	SRR36174754
RJNSPP24-013	Human	2022	ST19	cgST4454	SAMN48909855	SRR36174749
RJNSPP24-014	Human	2022	ST19	cgST4454	SAMN48909856	SRR36174748
RJNSPP24-018	Human	2022	ST19	cgST4454	SAMN48909857	SRR36174747
RJNSPP24-026	Human	2022	ST19	cgST291218	SAMN48909858	SRR36174746
RJNSPP24-027	Human	2022	ST19	cgST291218	SAMN48909859	SRR36174745
RJNSPP24-032	Human	2022	ST19	cgST4454	SAMN48909860	SRR36174786
RJNSPP24-034	Human	2022	ST19	cgST4454	SAMN48909861	SRR36174785
RJNSPP24-037	Human	2022	ST34	cgST52428	SAMN48909862	SRR36174784
RJNSPP24-038	Human	2023	ST34	cgST52428	SAMN48909863	SRR36174783
RJNSPP24-043	Human	2023	ST34	cgST52428	SAMN48909864	SRR36174782
RJNSPP24-046	Human	2023	ST19	cgST4454	SAMN48909865	SRR36174781
RJNSPP24-058	Human	2021	ST34	cgST52428	SAMN48909866	SRR36174780
RJNSPP24-059	Human	2021	ST34	cgST52428	SAMN48909867	SRR36174779
RJNSPP24-060	Human	2021	ST34	cgST52428	SAMN48909868	SRR36174778
RJNSPP24-061	Human	2021	ST34	cgST52428	SAMN48909869	SRR36174777
RJNSPP24-062	Human	2021	ST34	cgST52428	SAMN48909870	SRR36174775
RJNSPP24-063	Human	2021	ST34	cgST52428	SAMN48909871	SRR36174774
RJNSPP24-067	Human	2022	ST34	cgST52428	SAMN48909872	SRR36174773
RJNSPP24-068	Human	2022	ST34	cgST52428	SAMN48909873	SRR36174772
RJNSPP24-069	Human	2022	ST34	cgST52428	SAMN48909874	SRR36174771
RJNSPP24-070	Human	2022	ST34	cgST52428	SAMN48909875	SRR36174770
RJNSPP24-071	Human	2022	ST34	cgST52428	SAMN48909876	SRR36174769
RJNSPP24-075	Human	2022	ST34	cgST52428	SAMN48909877	SRR36174768
RJNSPP24-076	Human	2022	ST34	cgST52428	SAMN48909878	SRR36174767
RJNSPP24-077	Human	2022	ST34	cgST52428	SAMN48909879	SRR36174766
RJNSPP24-087	Human	2023	ST34	cgST17881	SAMN48909880	SRR36174764
RJNSPP24-091	Human	2023	ST19	cgST4454	SAMN48909881	SRR36174763
RJNSPP24-092	Human	2023	ST19	cgST4454	SAMN48909882	SRR36174762
RJNSPP24-093	Human	2023	ST19	cgST4454	SAMN48909883	SRR36174761
RJNSPP24-094	Human	2023	ST19	cgST4454	SAMN48909884	SRR36174760
RJNSPP24-095	Human	2023	ST19	cgST4454	SAMN48909885	SRR36174759
RJNSPP24-109	Human	2023	ST19	cgST4454	SAMN48909886	SRR36174758
RJNSPP24-115	Human	2021	ST19	cgST4454	SAMN48909887	SRR36174757
RJNSPP24-121	Swine	2022	ST34	cgST52428	SAMN48909888	SRR36174756
RJNSPP24-124	Wastewater	2022	ST19	cgST4454	SAMN48909889	SRR36174755
RJNSPP24-130	Wastewater	2023	ST19	cgST4454	SAMN48909890	SRR36174753
RJNSPP24-131	Wastewater	2023	ST19	cgST4454	SAMN48909891	SRR36174752
RJNSPP24-132	Wastewater	2023	ST19	cgST4454	SAMN48909892	SRR36174751
RJNSPP24-136	Wastewater	2023	ST19	cgST4454	SAMN48909893	SRR36174750

Table S2. Antimicrobial susceptibility profiles of *qnrS1*-positive isolates. The table presents MIC values and categorical interpretations for each antimicrobial class.

**Antimicrobial classes**		**Resistance**		**Intermediate**		**Susceptible**	
		**No.**	**%**	**No.**	**%**	**No.**	**%**
β-lactam	AMP	22	100.0	0	0.0	0	0.0
	IMI	1	4.5	0	0.0	21	95.5
	FOT	22	100.0	0	0.0	0	0.0
	AXO	22	100.0	0	0.0	0	0.0
	TAZ	22	100.0	0	0.0	0	0.0
	FOX	0	0.0	0	0.0	22	100.0
Tetracycline	TET	22	100.0	0	0.0	0	0.0
Chloramphenicol	CHL	22	100.0	0	0.0	0	0.0
Sulfonamide	SXT	0	0.0	0	0.0	22	100.0
Quinolone	NAL	1	4.5	0	0.0	22	100.0
	CIP	0	0.0	21	95.5	1	4.5
Aminoglycoside	GEN	1	4.5	0	0.0	21	95.5
	AMI	0	0.0	0	0.0	22	100.0
Macrolide	AZI	0	0.0	0	0.0	22	100.0
Polymixin	COL	0	0.0	22	100.0	0	0.0

AMP: ampicillin; IMI: imipenem; FOT: cefotaxime; AXO: ceftriaxone; TAZ: ceftazidime; FOX: cefoxitin; TET: tetracycline; CHL: chloramphenicol; SXT: trimethoprim/sulfamethoxazole; NAL: nalidixic acid; CIP: ciprofloxacin; GEN: gentamicin, AMI: amikacin; AZI: azithromycin; COL: colistin.
